# Novel *TBR1* c.1303C>T Variant Led to Diagnosis of Intellectual Developmental Disorder with Autism and Speech Delay: Application of Comprehensive Family-Based Whole-Genome Analysis

**DOI:** 10.3390/genes16020120

**Published:** 2025-01-22

**Authors:** Mario Ćuk, Busra Unal, Matea Bagarić, Goran Krakar, McKenzie Walker, Connor P. Hayes, Boris Gašpić, Goran Skular, Arezou A. Ghazani

**Affiliations:** 1 Department of Pediatrics, University Hospital Centre Zagreb, 10000 Zagreb, Croatia; 2 School of Medicine, University of Zagreb, 10000 Zagreb, Croatia; 3 Division of Genetics, Brigham and Women’s Hospital, Boston, MA 02115, USA; 4 Pediatric Clinic Sabol, 10000 Zagreb, Croatia; 5 SL Solutions, 10000 Zagreb, Croatia; 6 Department of Medicine, Brigham and Women’s Hospital, Boston, MA 02115, USA; 7 Harvard Medical School, Boston, MA 02115, USA

**Keywords:** *TBR1*, autism spectrum disorder, IDDAS, neurodevelopmental disorder

## Abstract

Background: Intellectual developmental disorder with autism and speech delay (IDDAS) is a rare and complex neurological disorder characterized by the presence of both intellectual and speech impairment and features of autism spectrum disorder (ASD). The prevalence of IDDAS is unknown but genetically, it is caused by heterozygous variants in the *TBR1* gene. Methods: A 7-year-old female with autistic features and delayed speech development was presented with unaffected parents. Trio-joint analysis was conducted on whole-genome sequencing (WGS) data from the proband and unaffected parents. A phenotype-driven analysis was conducted to investigate variants related to the patient’s clinical presentation. A zygosity-focused analysis was performed to investigate de novo and compound heterozygote variants related to the etiology. Results: The joint-genome analysis identified a novel NM_006593.4(*TBR1*):c.1303C>T p.Gln435* nonsense variant in the proband. The de novo analysis confirmed the absence of the variant in the parents. No additional causative variants were identified in genes associated with the proband’s phenotype. Conclusions: This is the first report of the NM_006593.4(*TBR1*):c.1303C>T variant in a patient with IDDAS. This study presents the clinical features of the patient and highlights details of trio-WGS analysis in the molecular diagnosis of this complex disease. Sharing these details is important, as they contribute to the understanding of the spectrum of this rare syndrome.

## 1. Introduction

Intellectual developmental disorder with autism and speech delay (IDDAS) is a broad-spectrum neurodevelopmental disorder that includes autism spectrum disorder (ASD). IDDAS is also characterized by varying degrees of intellectual disability (ID), language deficits, morphological abnormalities in the brain, and neurological and skeletal abnormalities [1,2,3,4]. While ASD is a multifactorial disorder involving many genes, IDDAS has a single-gene etiology (OMIM #606053). Heterozygous variants in T-box brain transcription factor 1 (*TBR1*) are known to cause IDDAS [1,2,3,4].

While the prevalence of IDDAS is unknown, ASD has a reported prevalence of approximately 1% [5,6,7]. ASD is characterized by qualitative impairment in social interaction and the presence of restricted repetitive motor and sensory behaviors [8,9]. As both syndromic and non-syndromic disorders, ASD is further specified based on the extent of involvement of language and intellectual development [7]. The clinical heterogeneity of ASD is due to complex genetic etiology and the involvement of many genes and loci [7]. Twin studies of ASD cases show 70–90% phenotypic concordance between identical twins, suggesting a genetic etiology for the disease. However, the underlying genetic cause of ASD has been identified only in 20–25% of the patients [10,11,12]. Contrary to ASD, IDDAS has a single gene etiology with heterozygous loss of function variants in the *TBR1* gene causing the disease [1,2,3,4].

Here, we describe the identification of a novel nonsense variant in a 7-year-old female with autistic features and neurodevelopmental abnormalities. A comprehensive trio-WGS analysis of the proband and unaffected parents identified a novel de novo likely pathogenic NM_006593.4(*TBR1*):c.1303C>T (p.Gln435*) variant. Given the complexity of IDDAS, sharing the genotype and phenotypical findings of IDDAS is important to understanding the spectrum of this rare syndrome.

## 2. Materials and Methods

### 2.1. Participants

The female index patient and her unaffected parents were included in the CROseq program at the Department of Pediatrics, University Hospital Centre Zagreb. CROseq is an international collaborative program between Brigham and Women’s Hospital (BWH) in Boston, USA, and the Department of Pediatrics at University Hospital Centre Zagreb in Croatia. The Mila za Sve Foundation in Rijeka, Croatia, funded the CROseq program. The participants provided their informed consent after the approved Institutional Review Board.

### 2.2. Clinical Assessment of Brain Activity by Electroencephalogram

An electroencephalogram (EEG) (Nihon Kohden, Neurofax EEG-1200K; Zagreb, Croatia) with a sampling rate between 200 and 10,000 Hz was performed to evaluate electrical brain activity. The patient’s forehead was cleaned with an alcohol swab before electrode placement to maintain the impedance of each electrode channel below 5 kΩ. According to FPz-F9 and FPz-AF7 of the international 10–20 system, electrodes were placed in a bipolar longitudinal montage and encompassed the brain of the same hemisphere.

### 2.3. Whole-Genome Sequencing

Whole-blood samples (2 mL) were obtained from the proband and her unaffected parents at University Hospital Centre Zagreb. Whole-genome sequencing (WGS) of the samples was conducted at the Medical College of Wisconsin in Milwaukee, USA. Genomic DNA was isolated at a 1.75–2.0 purity ratio and underwent robotic library preparation. The NovaSeq 6000 platform (Illumina, CA, USA), with a mean coverage depth of 40×, was utilized for sequencing.

### 2.4. Joint Trio-WGS Analysis

Trio-based whole-genome analysis was performed at BWH on the DNA obtained from the proband and her unaffected parents after ensuring satisfactory quality metrics of the WGS runs. The WGS quality metrics obtained from runs of the proband, mother, and father were, respectively, as follows: average variant depth: 47, 44, 42; homozygote/heterozygote ratio: 0.56, 0.57, 0.56; Ti/Tv ratio: 2.82, 2.81, 2.79; variant quality: 99%, 99%, 99%; number of SNPs (excluding low quality): 4,957,824, 493,395,9, 4,935,579; parental consanguinity check was passed; family relationship check was passed; uniparental disomy check was passed.

The Human Phenotype Ontology (HPO) terms of the proband’s phenotypical features informed the trio-WGS analysis and were as follows: Delayed speech and language development (HP:0000750), Autistic behavior (HP:0000729), and EEG with focal epileptiform discharges (HP:0011185). A comprehensive analysis encompassing variants from all genomic regions was conducted. After variant calling, the variant qualities were assessed based on variant allelic fraction and sequencing depth. High and medium confidence variants were included in further analysis. Failed and low-confidence variants were filtered out. The variant frequencies annotated from gnomAD (v2.1.1) were used in the population frequency assessment. The in silico prediction was performed by aggregating scores from Polyphen, SIFT, MutationTaster, Mutation Assessor, FATHMM, FITCONS, GENOCANYON, dbscSNV ADA, and dbscSNV RF. Splice site variants were assessed by the Splice AI prediction score. Variant prioritization was performed based on the genes’ relevance to the patient’s phenotype.

A de novo analysis was conducted to assess potentially causative variants related to the patient’s phenotype that were present in the patient but absent in both parents. A zygosity investigation was performed to identify compound heterozygous and homozygous variants related to the phenotype. This analysis was performed to assess the variants inherited from each unaffected parent related to the phenotype with an autosomal recessive mode of inheritance.

Variants were interpreted according to ACMG-AMP guidelines [13]. For PM2 and BS1 criteria, gene-specific thresholds were used to assess variants’ allelic frequency in gnomAD (v2.1.1). Aggregate in silico prediction score thresholds of 0.7 and 0.15 were assigned for deleterious and benign effects, respectively. For Splice AI, the upper threshold of 0.7 and a lower threshold of 0.2 were used. Intolerance to loss of function (LoF) was assessed by pLI = 1 and an observed/expected (O/E) score < 0.35. ClinVar submissions and online databases, such as PubMed, OMIM, Orphanet, and GeneReviews, were used to assess gene-phenotype relationships.

## 3. Results

### 3.1. Case Presentation

**Figure 1:**
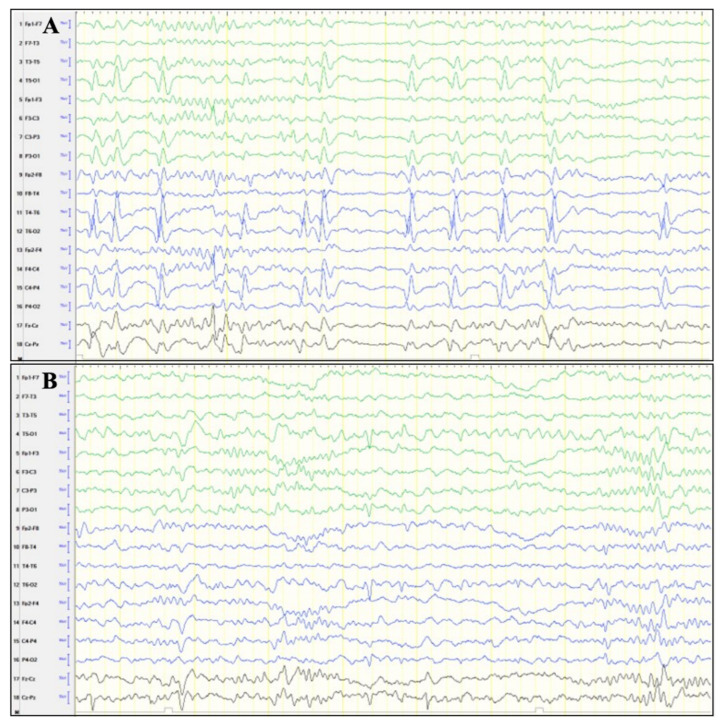
Electroencephalogram of the proband. (**A**) Sleep EEG at 1.5 years of age showing bilateral temporo-parieto-occipital focal epileptic discharges; (**B**) sleep EEG at 2.5 years of age showing only mild focal epileptic discharges representing significant improvement following antiepileptic therapy.

A 7-year-old female presented with delayed speech and language development, autistic behavior, and focal epileptic discharges. At birth, she was delivered at term by planned Cesarean section following an uneventful pregnancy. From early infancy, she was irritable, with sensory processing issues and sleep disturbances. At night, she would frequently cry inconsolably until becoming physically exhausted. In late infancy, she began selecting food according to its texture and started to refuse regular food. At 1.5 years of age, sleep EEG showed bilateral temporo-parieto-occipital focal epileptic discharges (Figure 1). Awake EEG showed only mild focal epileptic discharges. She did not present with clinical seizures.

At two years of age, she began displaying stereotyped behaviors, including hair pulling and repetitive dressing and undressing. She would either place the pulled hair in her mouth or twirl it around her fingers, and this behavior would occasionally escalate to her aggressively pulling out large chunks of hair. In kindergarten, she would play alone and would rarely communicate with others. She experienced severe temper tantrums and exhibited specific tactile aversions to various materials consisting of grass, sand, and plasticine. In a noisy environment, she would cover her ears with her palms. At 2.5 years, therapy with lamotrigine was initiated due to marked changes in her EEG. However, due to the progression of epileptic discharges, particularly during sleep, oxcarbazepine was introduced, with subsequent improvement in her EEG, still without manifestation of clinical seizures. Her brain MRI was normal.

Between the ages of 3 and 6 years, she would communicate by combining one or two words with a pointing gesture. Periodically, she would repeat all adopted words in her attempts to communicate. Towards the end of this period, she began using an assistive communication device. From 6 years of age, her sleep, sensory difficulties, and social skills improved. In familiar environments, she would use three-syllable grammatically incorrect sentences. In non-familiar environments, however, she would prefer using her assistive communication device. Undesirable behaviors observed earlier in life had become milder and were shorter in duration, appearing more consistent with manipulative behaviors. ASD in this patient was assessed per standard practice according to the ADOS-2 (The Autism Diagnostic Observation Schedule—Second Edition). A summary of the phenotype is provided in Table 1.

### 3.2. Trio-WGS Analysis

**Figure 2:**
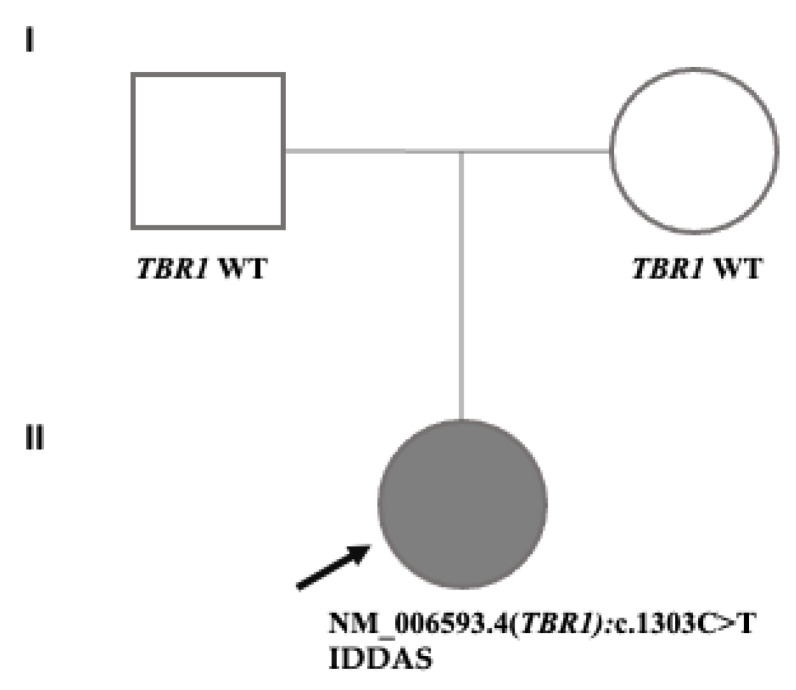
The family pedigree of the patient with the NM_006593.4(*TBR1*):c.1303C>T variant (**I**, **II**). Circles and squares denote female and male family members, respectively. The arrow shows the proband. The gray filling color represents IDDAS (intellectual developmental disorder with autism and speech delay). The *TBR1* genotype status for each participant is given under the circles or squares.

**Figure 3:**
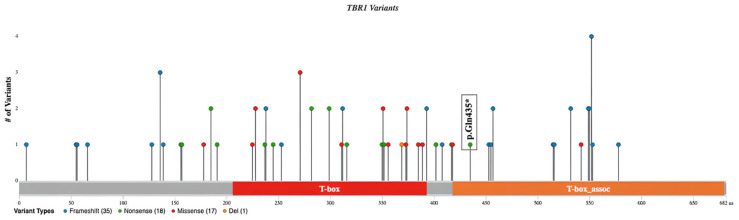
Lollipop plot of the deleterious *TBR1* variants reported in ClinVar and the published literature. The plot demonstrates the p.Gln435* variant detected in the proband in this study and frameshift and nonsense pathogenic *TBR1* variants reported in the ClinVar database and the published literature (n = 72). The Y axis indicates the variant number located at the given amino acid coordinate, and the X axis demonstrates the amino acid positions in the TBR1 protein. The red box shows the T-box domain and the orange box shows the T-box-associated domain.

Joint trio-WGS analysis was performed on the sequencing data obtained from the DNA of the patient and unaffected parents. The phenotype-based assessment interrogated 4,246,538 variants in 36,859 genes. The de novo interrogation evaluated the variants identified in the patient but were absent in the parents. The analysis revealed a novel de novo heterozygous NM_006593.4(*TBR1*):c.1303C>T (p.Gln435*) variant in the proband. (Figure 2). The *TBR1* gene (*604616) is diagnostically known to cause IDDAS with the established genotype-phenotype association in OMIM (#606053). No other deleterious variant in the genes associated with the patient’s phenotype (i.e., HPO list) was detected. *TBR1*:c.1303C>T is located at exon six at the T-box-associated domain in the transcript (Figure 3). This variant creates a premature termination codon (PTC) in the last exon of the *TBR1* in the T-box-associated domain. Notably, 17 pathogenic null variants have been reported in the last exon of *TBR1*, 12 of which are located downstream of *TBR1*:c.1303C>T(p.Gln435*). Given the PTC resulting from p.Gln435* is upstream to the PTC of these pathogenic null variants, the expected truncation protein resulting from p.Gln435* is likely pathogenic. LoF is a known mechanism of disease in *TBR1*, as indicated by its reported O/E score of 0.194 and pLI score of one. This variant has not been reported in ClinVar or any of the published literature. It is also absent in gnomAD, the largest publicly available population genome database. To account for the allele frequency in the Croatian-specific subpopulation, we investigated the presence of this variant in our internal CROseq database comprising 5 × 10^7^ variants from 1508 alleles obtained from 754 Croatian individuals. This variant was absent in the CROseq database. Therefore, this variant was interpreted as likely pathogenic using PVS1 and PM2 per ACMG guidelines.

The compound heterozygous analysis was performed to identify possible recessive variants related to the disease phenotype. The trio compound heterozygous analysis did not identify any such homozygous or compound heterozygous deleterious variant inherited from the parents.

## 4. Discussion

IDDAS (OMIM #606053) is defined as a neurodevelopmental disorder presenting with varying levels of intellectual disability, autism spectrum disorder, and language impairments. Clinical findings consist of moderate-to-severe speech delay, mild-to-severe developmental delay, intellectual deficiency, altered communication, stereotypies, behavior disorders (rituals, agitation, aggressive behavior, intolerance to frustration, selective eating disorder), sleep disorders (nocturnal awakening), and EEG abnormalities [3]. In some patients harboring pathogenic *TBR1* variants, structural brain abnormalities, neurological features (hypotonia, fine motor delay, abnormal movements, and epilepsy), and skeletal anomalies were also reported [14].

Heterozygous pathogenic *TBR1* variants are implicated in the etiology of IDDAS [1,2,3,4]. The *TBR1* gene is located at 2q24.2 and encodes a neuron-specific transcription factor of the T-box family [15]. This transcription factor is expressed in the deep cortex both during embryogenesis and adulthood [16]. *TBR1* has multiple roles in the cortical patterning of the central nervous system and neuronal development [15,17,18,19,20,21]. Reportedly, *TBR1* interacts with the CASK protein in the developing cerebral cortex. This interaction leads to the translocation of CASK to the nucleus, which in turn regulates *TBR1* target genes [1,2]. Additionally, *TBR1* regulates the expression of ASD-related genes, including *RELN*, *GRIN2B*, and *AUTS2* [1,22]. *TBR1* also interacts with key speech and language development genes, *FOXP1* and *FOXP2* [1,2,22].

The NM_006593.4(*TBR1*):c.1303C>T variant is located in exon 6. In ClinVar and the published literature, there are 72 reported pathogenic or likely pathogenic *TBR1* variants to date (21 December 2024) [2,14,23]. In total, 18 of these 72 variants are nonsense and 35 are frameshift. Given the pLI score of one, *TBR1* is considered intolerant to LoF. Therefore, variants that introduce a premature stop codon are expected to have a deleterious effect on proteins [14] and contribute to the mechanism of disease [2]. Of the pathogenic or likely pathogenic variants reported in ClinVar and the literature, 22 are located in exon 6, similar to our variant (Figure 3). No significant differences were observed in the type or severity of the clinical findings based on the variant’s location along the gene [14].

The comparison of phenotypes in reported *TBR1*-related IDDAS cases with frameshift and nonsense variants shows a wide clinical spectrum (Table 2). This includes ID (96%), ASD (88%), and global developmental delay, including language delay (96%) and motor delay (86%). Less frequently, EEG anomalies (46%) and seizures (14%) have been reported. Our patient exhibited ID, ASD, and speech delay. EEG recorded bilateral focal epileptic discharges localized to the temporal–parietal–occipital regions, a finding not previously reported in individuals with a nonsense variant in *TBR1*. One study describes a patient with a frameshift *TBR1* variant who presented with multiple focal epileptic discharges on both awake and sleep EEG, in addition to seizures, ASD, ID, and global developmental delay [24]. These findings are consistent with the EEG pattern observed in our patient and suggest that focal epileptic discharges may be part of the broader *TBR1*-related IDDAS phenotype. Further studies are required to establish a possible association between specific phenotypes and IDDAS-related genotypes.

In conclusion, we describe a novel de novo NM_006593.4(*TBR1*):c.1303C>T nonsense variant associated with IDDAS. Given the high clinical heterogeneity of IDDAS and the small number of studies that provide detailed phenotypes and genotypes of *TBR1*-related IDDAS, sharing rare genome findings with clinical descriptions is necessary to enhance our understanding of the genetic pathophysiology of IDDAS.

## Figures and Tables

**Table 1:** The clinical details of the proband.

Developmental Features		Psychological Features			Sleep Disorders	Brain MRI (Age)	Sleep EEG Features	Other Clinical Features
Speech Delay	DD/ID	Autistic Features	Altered Communication	Stereotypies			Focal Epileptic Discharges	
Moderate	Moderate	ADOS-2 Moderate	Present	Present	Nocturnal awakening	Normal (2 y)	Bilateral temporo-parieto-occipital	Food selectivity, specific tactile aversions (grass, sand, plasticine)

Abbreviations: DD: developmental delay; ID: intellectual disability; ADOS-2: Autism Diagnostic Observation Schedule—Second Edition.

**Table 2:** *TBR1* variants and their reported phenotypes in our patient and those reported in the literature.

Subject	Amino Acid Change	Effect	Inheritance	ASD	ID	Language Delay	Motor Delay	Seizures	EEG	Manuscript
1	p.Gln435*	nonsense	de novo	+	+	+	−	−	bilateral temporo-parieto-occipital focal epileptic discharges	current study
2	p.Trp271Arg	missense	de novo	+	+	+	+	+	N/A	[1]
3	p.Trp271Cys	missense	de novo	+	N/A	N/A	N/A	N/A	N/A	[1]
4	p.Lys389Glu	missense	de novo	+	+	+	N/A	−	N/A	[1]
5	p.Ala136Profs*80	frameshift	de novo	+	+	+	N/A	N/A	N/A	[2]
6	p.Lys228Glu	missense	de novo	+	+	+	N/A	N/A	N/A	[2]
7	p.Ser351*	frameshift	de novo	+	+	+	N/A	N/A	N/A	[2]
8	p.Gln178Glu	missense	inherited	+	−	+	N/A	N/A	N/A	[2]
9	p.Gln178Glu	missense	inherited	+	+	+	N/A	N/A	N/A	[2]
10	p.Gln418Arg	missense	inherited	+	+	+	N/A	N/A	N/A	[2]
11	p.Pro542Arg	missense	inherited	+	−	+	N/A	N/A	N/A	[2]
12	p.(Gly316Ter)	nonsense	de novo	+	+	+	+	−	normal	[3]
13	p.(Leu311Pro)	missense	de novo	+	+	+	+	−	N/A	[3]
14	p.(Thr532Argfs*144)	frameshift	de novo	+	+	+	+	−	N/A	[4]
15	p.(Thr532Argfs*144)	frameshift	de novo	N/A	+	+	+	N/A	N/A	[4]
16	p.(Tyr157*)	nonsense	N/A	+	+	+	+	−	normal	[14]
17	p.(Gln185*)	nonsense	de novo	+	+	+	+	−	not performed	[14]
18	p.(Ser238Thrfs*17)	frameshift	de novo	+	+	+	+	−	N/A	[14]
19	p.(Gln282*)	nonsense	de novo	+	+	+	+	−	N/A	[14]
20	p.(Trp299*)	nonsense	de novo	+	+	+	−	−	N/A	[14]
21	p.(Thr312Glnfs11*)	frameshift	de novo	+	+	+	+	−	normal	[14]
22	p.(Asp393Glyfs*2)	frameshift	de novo	+	+	+	+	−	normal	[14]
23	p.(Thr457Glnfs*30)	frameshift	de novo	+	+	+	−	−	slow pattern, asymmetric without paroxysmal activity	[14]
24	p.(Thr532Argfs*144)	frameshift	de novo	+	+	+	+	−	unspecific atypical diffuse α-like activity	[14]
25	p.(Thr532Argfs*144)	frameshift	de novo	+	+	+	+	−	N/A	[14]
26	p.(Thr532Argfs*144)	frameshift	de novo	+	+	+	N/A	−	normal	[14]
27	p.(Thr532Argfs*144)	frameshift	de novo	+	+	+	+	−	normal	[14]
28	p.(Thr532Argfs*144)	frameshift	de novo	−	+	+	N/A	−	normal	[14]
29	p.(Thr532Argfs*144)	frameshift	de novo	−	+	+	+	+	left temporal sharp and spike waves, diffuse arrhythmic slowdown	[14]
30	p.(Ser549Glyfs*128)	frameshift	de novo	+	+	+	+	+	epileptic activity in both hemispheres, disturbed background activity	[14]
31	p.(Pro550fs*127)	frameshift	de novo	+	+	+	+	−	N/A	[14]
32	p.(Gln552Alafs*122)	frameshift	de novo	−	+	+	+	−	abnormal	[14]
33	p.(Gln552Valfs*121)	frameshift	de novo	+	+	+	N/A	−	N/A	[14]
34	p.(Ile225Phe)	missense	de novo	+	+	+	+	+	right temporal spikes	[14]
35	p.Trp271Arg	missense	de novo	+	+	+	+	+	generalized multifocal seizures, focal epileptiform discharges in right frontal region	[14]
36	p.(Trp271Ser)	missense	de novo	−	+	+	−	−	N/A	[14]
37	p.(Val369_Ala371del)	in-frame deletion	de novo	+	+	+	+	−	not performed	[14]
38	p.(Gln373Arg)	missense	de novo	−	+	+	+	+	superimposed paroxysms of higher frequency, generalized with short spike-wave morphology in parieto-temporal regions	[14]
39	p.(Asn385Lys)	missense	de novo	+	+	+	−	−	paroxysmal rapid activity and polyspikes in the bilateral frontal region, high-amplitude sharp occipital waves	[14]
40	p.(Pro9Leufs*12)	frameshift	de novo	+	−	−	−	−	N/A	[25]
41	p.Phe124Valfs*18	frameshift	inherited	N/A	+	+	N/A	N/A	N/A	[17]
42	c.1129-1G>C	splice site	N/A	+	+	+	N/A	N/A	N/A	[26]
43	p.Tyr553Serfs*124	frameshift	de novo	+	+	+	+	N/A	N/A	[27]
44	(p.Gly533Leufs*143)	frameshift	N/A	+	+	+	+	+	multiple focal discharges in both awake and sleep stages; more pronounced in sleep	[24]

Abbreviations: ASD: autism spectrum disorder; EEG: electroencephalogram; ID: intellectual disability; N/A: not available.

## Data Availability

Data available upon request from the corresponding author.
